# Viral and Cellular Biomarkers in the Diagnosis of Cervical Intraepithelial Neoplasia and Cancer

**DOI:** 10.1155/2013/519619

**Published:** 2013-12-09

**Authors:** Maria Lina Tornesello, Luigi Buonaguro, Paolo Giorgi-Rossi, Franco M. Buonaguro

**Affiliations:** ^1^Molecular Biology and Viral Oncology, National Cancer Institute “Fondazione Pascale”, Cappella Cangiani, 80131 Naples, Italy; ^2^Servizio Interaziendale di Epidemiologia, AUSL Reggio Emilia, 42121 Reggio Emilia, Italy

## Abstract

Cervical cancer arises from cells localized in the ectoendocervical squamocolumnar junction of the cervix persistently infected with one of about 13 human papillomavirus (HPV) genotypes. The majority of HPV infections induces low grade squamous epithelial lesions that in more than 90% of cases spontaneously regress and in about 10% eventually progress to high grade lesions and even less frequently evolve to invasive cancer. Tumor progression is characterized by (1) increased expression of E6 and E7 genes of high risk HPVs, known to bind to and inactivate p53 and pRb oncosuppressors, respectively; (2) integration of viral DNA into host genome, with disruption of E2 viral genes and host chromosomal loci; and (3) molecular alterations of key regulators of cell cycle. Molecular markers with high sensitivity and specificity in differentiating viral infections associated with cellular abnormalities with high risk of progression are strongly needed for cervical cancer screening and triage. This review will focus on the analysis of clinical validated or candidate biomarkers, such as HPV DNA, HPV E6/E7 mRNA, HPV proteins, p16(INK4a) and Ki67, TOP2A and MCM2 cellular factors, and DNA methylation profiles, which will likely improve the identification of premalignant lesions that have a high risk to evolve into invasive cervical cancer.

## 1. Introduction

Cervical cancer is the third most common tumour in women worldwide with more than 85% of the cases occurring in low-to-medium-resource countries [1]. The introduction of tumour screening programs in many high-resource countries, over the past decades, has successfully decreased cervical cancer incidence and mortality [2]. Nevertheless, stable or even higher trends have been observed in countries where cervical screening is either absent or of low quality and low coverage [3, 4]. 

The role of human papillomaviruses (HPVs) in the aetiology of invasive cervical carcinoma has been well established. At least 13 genotypes of the alpha genus (HPV types 16, 18, 31, 33, 35, 39, 45, 51, 52, 56, 58, 59, and 68) have been found to be associated with the risk to develop cervical cancer and defined as “carcinogenic” viral types [5–7]. HPV16 is the most prevalent genotype in both squamous cell carcinoma (59.3%) and adenocarcinoma (36.3%) across the world [8]. HPV18, the second most common genotype, has been found in a higher proportion of adenocarcinoma (36.8%) compared to squamous cell carcinoma (13.2%) [8, 9]. Other oncogenic HPVs have a lower prevalence but still contribute to a significant fraction of cervical cancer [8]. HPV infection, on the other hand, is very common among young women with a peak of at least 20% among women aged between 20 and 24, and a subsequent decline to approximately 3% among women over 30 years of age [10, 11]. Thus, it is very relevant to identify biomarkers able to identify among persistently infected women those with a risk to develop cervical cancer.

The HPV lifecycle is characterized by infection of undifferentiated proliferating cells of the basal epithelial layer that become exposed through microwounds. HPV DNA episomes are maintained at low copy number in the nucleus and only early proteins are expressed from the viral genome. Differentiation of HPV-positive epithelial cells is accompanied by viral DNA replication and activation of the productive phase of the viral life cycle [12]. In particular, the expression of E6 and E7 genes, through the spinous and granular epithelial layers, deregulates cell cycle control inducing differentiating cells to enter into S phase and allowing amplification of the viral genome [12]. The late proteins L1 and L2 are actively expressed in the cornified layers where newly synthesized viral genomes are encapsidated and virions are shed [13]. The productive HPV infection could be clinically unapparent or associated with changes in the epithelial morphology leading to benign hyperproliferative flat warts, condylomata, or papillomas. In most women immune response to HPV infection develops after a period of months or years and results in adequate viral clearance [14]. The pattern of viral gene expression of high risk HPVs in low-grade squamous intraepithelial lesions (LSIL) is very similar to that seen in productive warts. Conversely, high grade intraepithelial lesions (HSIL) and invasive cancer represent abortive infections in which early genes E6 and E7, but not late genes L1 and L2, are expressed in all mucosal epithelial layers and the normal life cycle of the virus cannot be completed [15]. The abnormal constitutive expression of E6 and E7 seems to be a key event in malignant progression of infected cells and is associated with multiple alterations in viral and cellular pathways [16]. Although E6 and E7 proteins are consistently expressed in squamous intraepithelial lesions, only a subset of neoplastic lesions will persist and progress to invasive cancer suggesting that other molecular events are involved in cancer progression.

Integration of high risk HPV DNA into the host genome is also a crucial event in cervical carcinogenesis as it is found almost exclusively in high-grade lesions and invasive cancer often in association with progression and invasiveness [17]. Notably, HPV genomes have been shown to be integrated within the common fragile sites of the human chromosomes and into or close by cellular genes, such as VMP1, PVRL1, CHERP, CEACAM5, AHR, and MRF-2, in a significant number of HPV-related high grade, but not low grade, genital lesions indicating that this is a late and critical event in cancer progression [18, 19].

**Figure 1:**
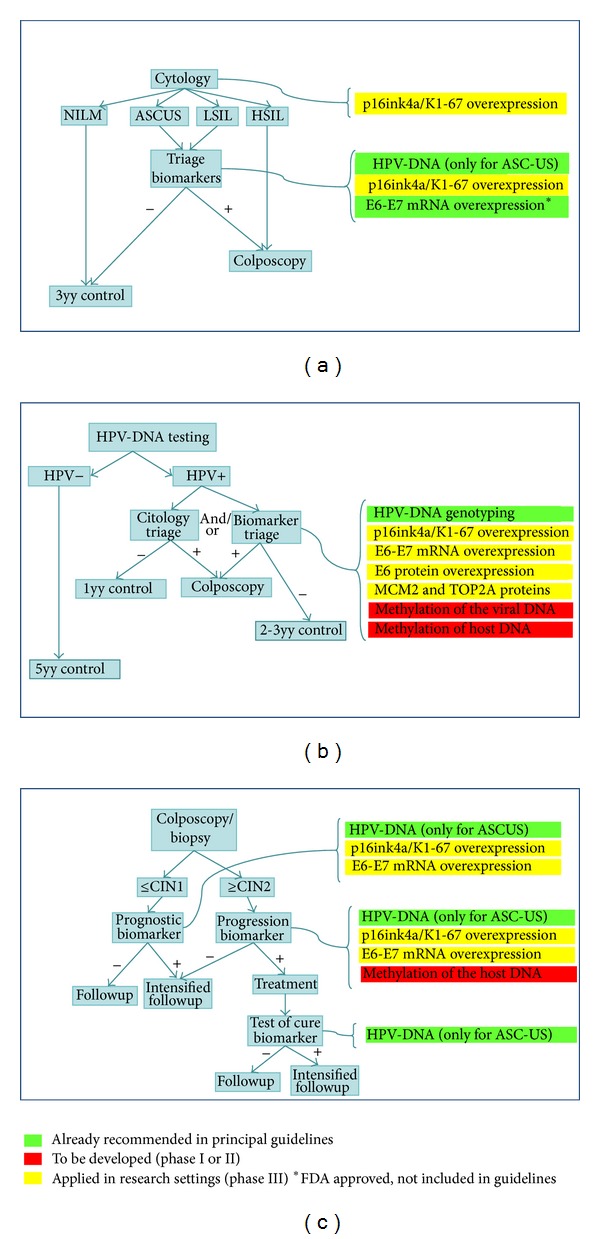
Flowchart describing the use of several biomarkers in different screening settings for cervical neoplasia. (a) Secondary prevention by means of screening based on cytology in conjunction with p16ink4a/Ki67 testing. Triage of equivocal cytology will take advantage of HPV tests and cellular biomarker-based assays. (b) Secondary prevention by means of screening based on HPV DNA tests. Progressing lesions of HPV-positive women will be identified by viral expression or cellular biomarker testing. (c) Triage of minor cytological cervical lesions and evaluation of recurrence after treatment for cervical precancer lesions with viral and cellular biomarkers. NILM = negative for intraepithelial lesion or malignancy; ASCUS = atypical cells of undetermined significance; SIL = squamous intraepithelial lesions.

Several biomarkers have been recognized which roughly identify specific stages in the natural history of HPV infection and cervical cancer progression. They include the presence of viral nucleic acids, viral proteins, or alteration of cellular factors induced by viral oncoproteins (Table 1). The flowcharts of possible clinical applications of available biomarkers in primary screening for cervical neoplasia and triage of women with HPV infection or equivocal/abnormal cytology are shown in Figure 1.

## 2. HPV DNA, RNA, and Proteins as Biomarkers for Cervical Neoplasia

Nowadays, numerous assays have been developed to detect nucleic acids of oncogenic and nononcogenic HPVs in cervical samples. The main advantage of using HPV testing is the high sensitivity, with a consequent high negative predictive value, since the absence of carcinogenic HPV indicates an extremely low risk of cervical intraepithelial neoplasia grade 3 (CIN3) or cancer [20–22], and the longer protection compared with cytology, since the risk of CIN3 and cancer remains very low up to 5 years after a negative HPV test [23]. The only concern is the low specificity of the HPV assays due to the fact that they cannot discriminate between transient and persistent HPV infections. Nevertheless, data from long-term prospective cohort studies and randomized clinical trials demonstrated that high risk HPV testing is highly sensitive and highly specific for detection of CIN2 or worse in women aged 30 years and older [24] and for diagnosis of adenocarcinoma, the precursors of which are often missed by cytological methods [25].

There are a wide range of commercially available HPV detection assays which are based on different techniques such as target amplification (mainly PCR), signal amplification, and probe amplification [26]. They can be divided in two groups according to (1) the ability to identify a pool of high risk HPV types, with or without genotypization of the most common high risk viruses (i.e., HPV16 and 18) or (2) to detect a broad spectrum of oncogenic and nononcogenic HPVs along with individual genotyping. While the assays of the first group are mainly used in screening programs, where there is no clinical benefit from the knowledge of specific HPV types, the assays of the second group are primarily used in HPV surveillance studies and to monitor the eventual spreading of particular viral types in vaccinated women.

### 2.1. HPV DNA Testing

Four HPV DNA assays have been approved by the Food and Drug Administration (FDA): (1) the Hybrid Capture 2 (Qiagen), detecting 13 high-risk HPVs; (2) Cervista HPV HR (Hologic), targeting 14 high-risk HPVs; (3) Cervista HPV 16/18 (Hologic), specifically designed to identify HPV16 and 18; and (4) Cobas 4800 HPV (Roche Diagnostics), targeting 14 high-risk HPVs (Table 1). Hybrid Capture 2 is a solution-phase hybridization assay and signal amplification that detects chemiluminescence by using specific HPV probes targeting 13 carcinogenic HPVs, specifically genotypes 16, 18, 31, 33, 35, 39, 45, 51, 52, 56, 58, 59, and 68 [27]. Hybrid Capture 2 test was approved by the FDA in 1999 for the triage of women with atypical squamous cells of undetermined significance (ASCUS), and in 2003 for primary cervical screening in conjunction with cytology. Arbyn et al. performed a meta-analysis of 39 studies and found that the pooled sensitivity of Hybrid Capture 2 was 90.4% (95% confidence interval [CI]: 88.1–92.3%) and 93.7% (95% CI: 90.4–95.9%), whereas the pooled specificity was 58.3% (95% CI: 53.6–62.9%) and 52.3% (95% CI: 45.7–58.7%) for predicting presence or absence of CIN2 or CIN3 or worse, respectively, in women with a cytological diagnosis of ASCUS [21]. In primary screening, the pooled sensitivity of Hybrid Capture 2 in European and North American studies was 96% (95% CI: 95–98%) for CIN2 or worse whereas the pooled specificity was 91% (95% CI: 90–93%) [21]. Cervista HPV HR and COBAS 4800 HPV tests were approved by the FDA in 2009 and 2011, respectively, for the triage of women older than the age of 21 years with diagnosis of ASCUS and for determining the presence of carcinogenic HPV types in conjunction with cytology in women older than 30 years. The relative accuracy of Cervista HPV and COBAS 4800 HPV versus the Hybrid Capture 2 assay to find underlying CIN2 or CIN3 or worse have been evaluated in primary screening studies [28, 29]. Compared with Hybrid Capture 2 the relative sensitivity of Cervista HPV and COBAS 4800 HPV was 0.97 (95% CI: 0.93–1.02) and 0.98 (95% CI: 0.88–1.10), respectively, while their relative specificity was 1.03 (95%CI: 1.02–1.04) and 1.00 (95%CI: 0.98–1.03), respectively.

In respect to the accuracy of Hybrid Capture 2 to find CIN2 or worse in the triage of women with low SIL, the meta-analysis of 24 studies showed that the pooled absolute sensitivity was 95.4% (95% CI: 94.0–96.5%) and 96.4% (95% CI: 90.5–98.7%), whereas the pooled specificity was 27.8% (95% CI: 23.8–32.1%) and 23.7% (95% CI: 19.4–28.7%) for the outcomes of CIN2 and CIN3 or worse, respectively. Similar to Hybrid Capture 2, the sensitivity of most of the other assays targeting the DNA of all 13 high risk HPV DNAs was high (>90% for CIN2 and CIN3), whereas the specificity was low in low SIL triage [21]. HPV DNA assays targeting a limited number of oncogenic viruses, including Cervista HPV 16/18, have a significant lower sensitivity but much higher specificity for the detection of high grade lesions in the triage of women with ASCUS or low SIL due to the fact that these assays only detect the subset of the most common carcinogenic HPV types in cervical neoplasia [21]. The HPV16 and HPV18 genotyping, for its high specificity, have been included in the US guidelines for the triage of HPV positive and cytology negative women [30, 31].

Several other HPV tests targeting at minimum the 13 high risk HPVs have been clinically evaluated, following the strategy reported by Meijer et al. [32], and the absolute as well as the relative accuracy for the detection of CIN2 or worse was very similar to that of Hybrid Capture 2 assay in screening populations [21, 22].

The U.S. Preventive Services Task Force (USPSTF) and a multidisciplinary partnership among the American Cancer Society/American Society for Colposcopy and Cervical Pathology/American Society for Clinical Pathology (ACS/ASCCP/ASCP), as well as several health authorities in Europe (HTA Italia, Health council Netherland) [33, 34], recommended HPV DNA test as a primary cervical cancer screening method. Some European countries [33, 34] adopted the Meijer et al. criteria for clinical validation of the HPV DNA test. The Italian HTA report referred 5 validated tests up to November 2011: (1) the Hybrid Capture 2 (Qiagen); (2) Cervista HPV HR (Hologic); (3) Cobas HPV (Roche Diagnostic); (4) PapilloCheck (Greiner-BioOne); and (5) Abbott real-time high risk HPV.

### 2.2. HPV RNA Testing

HPV RNA assays are designed to detect viral mRNAs encoding for the E6 and E7 proteins which are the most critical factors for the development of cervical cancer. Overexpression of HPV E6 and E7 mRNAs has been evaluated as a marker for the transition from a productive infection to an abortive infection that eventually promotes cell transformation. Four assays are currently available for detection of HPV E6/E7 mRNA in cervical samples. The APTIMA (GenProbe) and OncoTect (IncellDX) assays are based on reverse transcriptase (RT) and PCR technique and detect E6/E7 mRNA from 14 and 13 high risk HPV genotypes, respectively. The PreTect HPV-Proofer (Norchip) and NucliSENS EasyQ (Biomerieux), both relying on nucleic acid sequence-based amplification (NASBA), are able to detect E6/E7 transcripts from the five most common high-risk viral types in cervical carcinoma (HPV 16, 18, 31, 33, and 45). APTIMA was approved by FDA in 2011 for the triage of women with ASCUS cytology and older than the age of 21 years or for screening of women of 30 years of age and older in conjunction with cytology. Several studies have compared the APTIMA to Hybrid Capture 2 and cytology-based tests for both primary cervical cancer screening as well as triage of ASCUS or low SIL. A recent meta-analysis showed that testing for viral RNA with APTIMA assay in the triage of women with ASCUS was as sensitive but more specific for identifying CIN2 or worse compared with Hybrid Capture 2 (specificity ratio of 1.19 (95% CI: 1.08–1.37)) [35]. However, testing for viral RNA with APTIMA was significantly more specific than Hybrid Capture 2 (ratio of 1.37; 95% CI: 1.22–1.54) without losing sensitivity (ratio of 0.96 for CIN2 and 0.98 for CIN3 or worse with 95% CIs including unity for both outcomes) in the triage of low SIL [35]; information about long-term protection, that is, longitudinal sensitivity, of the mRNA based tests are lacking. Moreover, the The PreTect HPV-Proofer and NucliSENS EasyQ, identifying the RNA of HPV16, 18, 31, 33, and 45, showed significantly lower sensitivity but higher specificity compared to Hybrid Capture 2 in both the triage of ASCUS and low SIL [21].

### 2.3. Testing for HPV Proteins

Two HPV protein assays are available to detect protein levels in cervical cell exfoliates, namely, the OncoE6 (Arbor Vita Corporation) and Cytoactiv (Cytoimmun Diagnostics). The OncoE6 is able to identify the E6 protein encoded by HPV 16, 18, and 45. A pilot study performed with OncoE6assay showed that the expression of E6 from HPV 16, 18, and/or 45 may be more specific for the detection of CIN3 or worse compared with HPV-DNA tests [36]. Clinical validation of this assay is ongoing in a population-based study in China, and preliminary results suggested that the OncoE6 has better specificity than Hybrid Capture 2 (98.9% versus 86.8%, resp.) but lower sensitivity (67.3% versus 98%, resp.) for the detection of CIN3 or worse [37]. Given the increased specificity, the assay may be a useful tool in the triage of HPV positive women. In addition, due to the limited laboratory equipment needed for the assay, it may have applications in low-resource settings. The Cytoactiv assay is designed to measure the loss of the expression of L1 which has been suggested as a marker of progressive lesions [38, 39]; however, the clinical significance of such analysis remains to be determined.

## 3. Cellular Biomarkers in Cervical Cancer

The functional inactivation of p53 and pRb oncosuppressors by E6 and E7 oncoproteins determines the alteration of several cellular pathways relevant for cell transformation and cancer development. The expression of E7 determines the inactivation of pRb with a consequent increase of free E2F in the cell, leading to both an increase of cyclin-dependent kinase inhibitor p16 (p16INK4a) and aberrant proliferation (marked by increased levels of Ki-67 expression) [40, 41]. Therefore, p16 overexpression, identified by immunostaining or enzyme-linked immunosorbent assay (ELISA), can be considered as a marker of HPV infection and of activated expression of viral oncogenes and virus-induced cell cycle deregulation [42–44].

The use of p16INK4a immunohistochemistry is also a very important tool for the improvement of the diagnostic accuracy, reliability and quality in histopathology of cervical lesions [45]. The accuracy of p16INK4a testing has been also evaluated in the triage of ASCUS and low SIL cytology [46]. A large meta-analysis including seventeen studies showed that the pooled sensitivity of p16INK4a to detect CIN2 or worse was 83.2% (95% CI, 76.8%–88.2%) and 83.8% (95% CI, 73.5%–90.6%) in ASCUS and low SIL cytology, respectively, and the pooled specificities were 71% (95% CI, 65%–76.4%) and 65.7% (95% CI, 54.2%–75.6%), respectively [47]. Studies based on both Hybrid Capture 2 and p16INK4a testing showed that they had similar sensitivity, but p16INK4a has a statistical significant higher specificity in the triage of women with ASCUS (relative sensitivity, 0.95 (95% CI, 0.89–1.01); relative specificity, 1.82 (95% CI, 1.57–2.12)). In the triage of low SIL, p16INK4a has significantly lower sensitivity but higher specificity compared with Hybrid Capture 2 (relative sensitivity, 0.87 (95% CI, 0.81–0.94); relative specificity, 2.74 (95% CI, 1.99–3.76)), [47]. Moreover, overexpression of p16INK4a has been shown to be a useful marker for CIN2 or to predict development of CIN2 within 3 years among HPV positive women, especially those aged 35–60 years. Carozzi et al. reported that CIN2 or worse was detected in a higher number of p16INK4a-positive women (8.8% (95% CI, 5.8–11.8)) than in negative women (3.7% (95% CI, 1.9–5.4)) during the followup. CIN3 or worse was detected more frequently in p16INK4a-positive women (4.4% (95% CI, 2.3–6.6)) than in negative women (1.3% (95% CI, 0.2-2.3)) during followup [48].

The proliferation antigen K1-67, which is expressed during the G2 and mitotic phases of the cell cycle, has been demonstrated in many studies to be a reliable indicator of the growth fraction of a tumor. Reuschenbach et al. analyzed a series of 138 cervical cone biopsies and showed that p16INK4a and Ki-67 were coexpressed in dysplastic lesions only [49]. A dual p16/Ki-67 immunocytochemistry assay is now also available for use as an adjunctive test in cervical cancer screening (CINtec Plus, mtm laboratories). The sensitivity of p16INK4/Ki67 dual stain (CINtec Plus) cytology has been evaluated in 776 retrospectively collected ASCUS/LSIL cases and found to be of 92.2% in ASCUS and 94.2% in low SIL, while specificity rates were 80.6% (ASCUS) and 68.0% (low SIL), respectively [50]. Similar sensitivity and specificity profiles were found in women aged <30 years versus women aged >30 years. Thus, dual-stain cytology showed comparable sensitivity but significantly higher specificity compared to testing for high risk HPVs and for p16INK4 alone. The p16INK4/Ki67 dual stain was also tested in a very large prospective clinical trial performed in five countries across Europe, which enrolled 27,456 women in a screening setting. Results obtained with conventional cytology, HPV (Hybrid Capture 2), and p16INK4a/Ki67 dual-stained cytology showed that p16INK4a/Ki-67 dual stained cytology testing significantly increased the sensitivity for diagnosis of CIN2 or worse over cytology while maintaining an high specificity.

Recent studies have shown that minichromosome maintenance protein 2 (MCM 2) and topoisomerase II alpha (TOP2A) proteins are expressed in cells with aberrant S-phases and including HPV-transformed cells in association with elevated expression of the HPV E6/E7 proteins [51, 52]. The ProExCTM assay developed by Becton-Dickinson is based on an antibody cocktail recognizing both MCM2 and TOP2A proteins. In a direct comparative study with the p16INK4a testing, the BD ProExCTM marker panel revealed a higher sensitivity for detecting women with low SIL but showed less specificity to identify cases with high SIL [53, 54]. Moreover, the use of BD ProExC assay for the triage of women testing positive for high risk HPVs was found to increase the specificity (98.3% versus 85.0%) and the positive predictive value (41.7% versus 9.3%) of the screening compared to the high risk HPV test alone [55].

The E6 proteins of oncogenic HPVs have been shown to promote the transcription of telomerase reverse transcriptase (TERT) which stabilizes and repairs the repeated DNA sequences at the telomere end of chromosomes [56]. Gain of chromosome 3q, containing the sequence for the telomerase RNA component (TERC), and gain of chromosome 5p, containing the TERT gene, have been associated with CIN2 or worse in cervical tissue biopsies, with a specificity of 97% [57–59]. The evaluation of gain of chromosomes 3q and 5p with fluorescence in situ hybridization (FISH) and multiplex ligation-dependent probe amplification (MLPA) may be a useful marker for the identification of progressing lesions. The analysis of TERT and TERC copy number increase, however, is limited in cytological samples due to the presence of predominately normal cells in these specimens [60].

Emerging evidence suggests that microRNAs (miRNAs), small noncoding single-stranded RNAs that regulate cell gene expression, might be involved in the pathogenesis of several human cancers, including cervical carcinoma [61]. Several miRNAs, such as miR-9, miR-127, miR-145, miR-146a, miR-199a, miR-200a, and miR-424, have been found dysregulated in cervical carcinoma [62, 63]. Li et al. [64] reported that among 171 women with CIN miR-218 levels were lower in patients with high-risk HPV than in those with low-risk or intermediate-risk HPVs. Wang et al. [65] observed that the expression of miR-375 in 170 cervical cancer tissues was significantly decreased compared with 68 normal tissues suggesting that downregulation of miR-375 could be involved in the progression of cervical cancer [65]. Therefore, miRNA deregulation may play an important role in cervical cancer progression and the evaluation of specific miRNAs could represent new candidate markers for cancer screening and prognostic evaluation of patients with cervical neoplasia.

## 4. Viral and Cellular Gene Methylation in Cervical Neoplasia Progression

DNA methylation is one of the epigenetic mechanisms that influence gene transcription, chromatin structure, genomic stability, and the inactivation of imprinted genes and X chromosome [66]. Abnormal methylation of promoters of tumour suppressor genes is common in various cancers, and the analysis of DNA methylation as a biomarker in clinical oncology seems to be promising [67]. Recent studies have shown that methylation of viral and cellular DNA is a potential biomarker for the improved accuracy of cervical screening and for the triage of abnormal cytology or high-risk HPV-positive women [37]. The direct relationship between methylation status of HPV L1 gene and diagnosis of CIN2 seems to be relatively consistent in most studies; however, the association between methylation in the upper regulatory region of HPV genomes and CIN2 is controversial given that some studies found decreased methylation of CpG sites within the HPV regulatory region while other studies report an increased methylation in such viral region. Two studies have recently described the comprehensive analysis of whole-genome methylation patterns of HPV16, HPV31, HPV18, and HPV45 in a greater number of specimens from a large cohort study [68, 69]. The investigators found that elevated levels of DNA methylation on multiple CG sites in the L1, L2, E2, and E4 ORFs were significantly associated with CIN2 or worse after accounting for multiple testing [68, 69]. These initial data are promising and may demand for the development of a commercial HPV DNA methylation test to be used in the triage of HPV positive women. However, the importance of quantitative measurement of HPV DNA methylation needs to be validated in larger studies in diverse clinical settings.

DNA methylation of several human genes has been shown to be also a relevant event for cervical carcinoma development. The treatment of HPV-positive cervical cancer cell lines with demethylating agents, coupled to expression microarrays, has allowed the identification of genes encoding for SPARC and TFPI2 as highly methylated in invasive cervical cancer [70]. Another approach based on the restriction landmark genomic scanning (RLGS) allowed the identification of genes encoding for NOL4 and LHFPL4 as methylated in cervical cancer [71]. The use of differential methylation hybridization (DMH) using a pilot methylation array allowed the identification of SOX1, NKX6-1, PAX1, WT1, and LMX1A as frequently methylated genes in cervical cancer and precursor lesions [72]. Moreover, quantitative DNA methylation analysis of these genes demonstrated the possibility of using them to detect CIN3 and worse lesions from cervical scrapings [73]. The methylation status of numerous other genes has been analysed in cervical cancer; among these 15 genes has been evaluated in five or more studies and only three of them (DAPK1, CADM1, and RARB) were found with a consistent elevated methylation in cervical cancer across studies [74]. Nevertheless, several very sensitive and specific methods have been developed to detect gene methylation such as quantitative methylation-specific polymerase chain reaction. These techniques together with the fact that aberrant methylation can be detected in cervical smears up to seven years prior to the diagnosis of cervical cancer strongly suggest that host gene methylation analysis may be a valuable strategy for the triage of women positive for high risk HPVs [75]. Moreover, methylation of certain genes is more specific for cervical adenocarcinoma (in situ) and its detection in cervical scrapings can therefore guide for appropriate therapy [74].

## 5. Conclusions

Testing for nucleic acids of high risk HPVs or for cellular surrogate markers of HPV transforming processes will most probably be the primary cervical cancer screening method in many countries. HPV-based tests offer a more sensitive way to identify women with high-grade cervical disease compared with cytology-based methods and in the future specific HPV testing will be very important for screening, for triage of women with abnormal cytology, and for viral surveillance and monitoring of vaccine efficacy. Many commercially available assays have been already approved by FDA or have been clinically validated in accordance with international approved validation guidelines. In general the large majority of available assay have high sensitivity and specificity for the identification of CIN2. But the majority of the CIN2 and many CIN3 lesions regress and it would be very relevant to identify biomarkers that identify the minority of high grade CIN lesions that will not regress. Unfortunately, CIN2 and CIN3 are the endpoint of all the available studies and the target of treatment; consequently we can have only indirect evidence for prognostic biomarkers able to identify high grade lesions that could be not treated.

New biomarkers such as viral and cellular methylation profiles could represent the most accurate markers for cancer progression. Nevertheless, results on the novel promising biomarkers are in general based on small sample size, and additional clinical trials are needed to determine the true clinical value of these new assays.

## Figures and Tables

**Table 1:** Commercial available assays targeting viral as well as cellular biomarkers.

Available assays	Manufacturer	Target	HPV genotypes	Genotyping	FDA approved
Viral Assay HPV DNA					
COBAS 4800	Roche	L1 DNA	13 HR HPV and HPV66	16 and 18	Yes
Cervista	Hologic	L1 DNA	13 HR HPV and HPV66	16 and 18	Yes
Hybrid Capture 2	QIAGEN	Full Genome	13 HR HPV and HPV66	No	Yes
Amplicor	Roche	L1 DNA	13 HR HPV	No	No
CareHPV	QIAGEN	L1 DNA	13 HR HPV and HPV66	No	No
Digene HPV eHC	QIAGEN	Full Genome	13 HR HPV, HPV66 and 82	No	No
EIA kit HPV GP HR	Diassay	L1 DNA	13 HR HPV and HPV66	No	No
INFINITI HPV-HR QUAD	AutoGenomics	E1 DNA	13 HR HPV and HPV66	No	No
RT HPV	Abbott	L1 DNA	13 HR HPV and HPV66	16 and 18	No
Digene HPV eHC 16 18/45	QIAGEN	Full Genome	13 HR HPV, HPV66 and 82	16, 18, and 45	No
Clart	Genomica	L1 DNA	13 HR HPV and 22 no HR	Yes	No
INFINITITM	Genomica	L1 DNA	13 HR HPV and 12 no HR	Yes	No
InnoLiPA	Innogenetics	L1 DNA	13 HR HPV and 15 no HR	Yes	No
Linear Array	Roche	L1 DNA	13 HR HPV and 24 no HR	Yes	No
Multiplex HPV genotyping	Multimetrix	L1 DNA	13 HR HPV and 11 no HR	Yes	No
PapilloCheck	Greiner Bio-One	E1 DNA	13 HR HPV and 11 no HR	Yes	No
HPV RNA					
APTIMA	GenProbe	E6/E7 mRNA	13 HR HPV and HPV66	No	Yes
NucliSens EasyQ	Biomerieux	E6/E7 mRNA	5 HR HPV	16, 18, 31, 33, and 45	No
OncoTect	IncellDx	E6/E7 mRNA	13 HR HPV	Yes	No
PreTect Proofer	Norchip	E6/E7 mRNA	5 HR HPV	16, 18, 31, 33, and 45	No
HPV Proteins					
Cytoactiv	Cytoimmun Diagnostics	L1	All known HPVs	No	No
OncoE6	Arbor Vita	E6	3 HR HPV	16, 18, and 45	No
Cellular Assay					
CINtec	mtm Laboratories	p16ink4a			No
CINtec Plus	mtm Laboratories	p16ink4a/K1-67			No
Ki-67 (MIB1)	DakoCytomation	Ki-67			No
ProEx C	Becton Dickinson	TOP2A/MCM2			No

## Abbreviations

HPV: Human papillomavirus
ICC: Invasive cervical carcinoma
SIL: Squamous intraepithelial lesion
ASCUS: Atypical squamous cells of undetermined significance
NILM: Negative for intraepithelial lesion and malignancy.
